# Improvised use of a digital tool for social interaction in a Norwegian care facility during the COVID-19 pandemic: an exploratory study

**DOI:** 10.1186/s12913-022-07526-0

**Published:** 2022-02-01

**Authors:** Abeer Badawy, Mads Solberg, Aud Uhlen Obstfelder, Rigmor Einang Alnes

**Affiliations:** 1 Department of Health Sciences in Ålesund, Faculty of Medicine and Health Sciences, Norwegian University of Science and Technology, Larsgårdsvegen 2, 6009 Ålesund, Norway; 2 Center for Care Research, Department of Health Sciences in Gjøvik, Faculty of Medicine and Health Sciences, Norwegian University of Science and Technology, Teknologivegen 22, 2815 Gjøvik, Norway

**Keywords:** Interactive technology, Caring practices, Care facilities, Pandemic, Social communication

## Abstract

**Background:**

Digital tools for social communication have been deployed in care facilities during the COVID-19 pandemic to facilitate social connectedness between older people and their next of kin in a safe manner. This study explores how and why health care professionals facilitate the ad hoc and prompt use of a technology for social communication, known as KOMP, in care facilities in western Norway to promote communication and social engagement among residents and their next of kin during the crisis.

**Methods:**

To investigate the perspectives and practices of health care professionals, we conducted focus groups, individual interviews, and participant observation in public short- and long-term care facilities in western Norway. An explorative investigation with inductive content analysis was applied to analyse interview transcripts and fieldnotes from participant observation.

**Results:**

The resulting qualitative data reveal that prompt implementation of interactive technology to cope with social distancing during the pandemic added new routines to the staff workload. Using this interactive technology entailed new forms of collaborative work among residents, next of kin, health care professionals and technology facilitators. Additionally, the staff articulated a sense of responsibility towards using KOMP as a meaningful and practical tool for social communication in an extraordinary period of reduced social contact.

**Conclusions:**

Improvised implementation of KOMP as an interactive technology shapes work routines, introduces new tasks and creates additional responsibilities. Despite creative efforts by health care staff, however, using KOMP remains constrained by the physical and cognitive abilities of its users. We suggest that health care managers ask a deceptively simple question when introducing novel technologies in health care contexts, namely: what kind of invisible work do these devices entail?

## Background

A growing number of technology-based interventions are used to support the health and quality of life of residents in care facilities. The onset of COVID-19 and the ensuing social distancing policies have led to a burgeoning interest in technology-based solutions, including digital devices such as tablets, wearable devices, and digital communication platforms to provide care and promote health [1]. Indeed, during the COVID-19 pandemic, several digital technologies have been adopted as alternatives to face-to-face communication [2]. To reduce loneliness and social isolation, many long-term care facilities have provided technologies such as Skype, FaceTime, and Zoom to allow residents to interact digitally with their relatives [3–5].

Previous studies on the feasibility of using digital interactive technologies among elderly individuals document the promises and challenges of these technologies [6, 7]. Siniscarco et al. [8] concluded that communication through video conferencing, when in-person visits are limited or impossible, may benefit residents who are distanced from close family or friends to reduce social isolation and mitigate loneliness. Applications of mobile technologies could also help older adults stay connected to friends and family, remain active, and access resources to address their physical and mental health needs [9]. Video-call interventions, such as a system known as ‘Skype on Wheels’, have been devised to support older people in care environments to better connect with their families. Such technology, however, requires continuous adjustments to accommodate characteristic features at each site of intervention to overcome barriers and maximize engagement from users [10]. Another recent trial, in a Norwegian context, introduced tablet computers (iPads), based around one-to-one tutoring, to facilitate social engagement among older adults in care facilities [11]. Participants reported satisfaction with these devices, and the authors observed an increase in social participation through communication apps, such as messaging and video conversations.

Nevertheless, routine use of digital communication tools in health care services in general, particularly in nursing homes, is challenging due to organizational, cultural, technological, and ethical issues. Some elderly individuals also struggle with digital technologies due to physical and cognitive impairments, low digital literacy, and social barriers [12]. There are also factors endogenous to care facilities that may prevent rapid adoption of novel technology in such institutions. For example, a strenuous workload and other practical constraints in the social ecology of care facilities preclude acceptance of technology by residents, their next of kin, and health workers. Other concerns include staff turnover due to rotation of health care professionals between departments and short-term contracts that require new training and a lack of adequate learning arrangements for making use of new digital tools. Disruptions of existing workflows, a lack of defined roles, and negative sentiments about technology are other barriers to the adoption and implementation of digital interventions in care [13].

The COVID-19 pandemic offers a unique opportunity to explore how a novel tablet-like device known as KOMP for promoting social communication affects practices of care. Designed to facilitate communication between users and next of kin in a simple and safe way, KOMP was adopted to support digital communications by many care facilities in Norway, beginning in March 2020. This *ad hoc* implementation of a device for corresponding with next of kin was necessitated by enforcement of social distancing and visitation restrictions. In contrast to many technologies, whose use in health care contexts is carefully planned, the prompt use of KOMP did not appear to be associated with comprehensive plans due to the necessity to maintain social communication between residents and their next of kin during mandatory social distancing. Data collection during a period characterized by exceptional restrictions on social life in health care facilities therefore offered an occasion to explore how people use this technology to promote social communication and interaction between residents and their next of kin, who could not visit as usual. Additionally, this case illustrates how the intensified use of interactive technology altered work practices within the care facility.

In a classic study of socially situated technology, Akrich [14] invoked the notion of a “script” to conceptualize how designers and product developers build assumptions about the world, including social practices, values, and cultural beliefs, into the “technical contents” of new devices. Like a film script, technical objects have intended uses and meanings, which define “a framework of action, together with the actors and the space in which they are supposed to act” [14, p. 208]. A metaphor drawn from the performing arts; the notion of a script is also useful for analysing new technologies in contexts such as health care. This concept implies that actors frequently adapt existing scripts or fashion new scripts that are suitable for the practical context in which a technology is applied. In Akrich’s terms, a script is dynamic, and it can be fine-tuned to various applications. This process, whereby scripts, as a framework for action, are repurposed by different stakeholders in novel situations, is known as “re-scripting” [14]. While a technological script by itself does not determine the actual use and distribution of roles with respect to a given technology [15], users frequently adapt existing scripts according to their goals. This can be understood as a negotiation process between technology, users, and different use contexts [16]. Moser [17], for instance, explored the social consequences of active-assisted living (AAL) devices through the lens of “re-scripting” by analysing models of videoconferencing consultations in patients’ homes. These practices require new collaboration patterns and involvement of professionals in both municipal health care and specialist care.

In our study, we employ this framework to explore how KOMP performs as an interactive technology for social communication in care facilities. While the main users of this “one-button computer” are usually elderly people who live at home but want to connect with family and friends, the use of KOMP in care facilities requires a productive interplay between a wider cast of characters, which includes relatives, health care professionals, and technology facilitators.

To the best of our knowledge, there is a descriptive report (in Norwegian) that deals with experiences of elderly individuals with cancer who use KOMP to counter loneliness [18], and refined and theory-driven analysis of the use of KOMP to bring people together and reduce loneliness among older adults [19]. Additionally, there are few studies describing the implementation of interactive technology devices for social communication between older people and their next of kin in care facilities [20]. In our study, we ask how and why health care professionals facilitate communication and social engagement between older people and their next of kin in care facilities using interactive technologies. Through the conceptual lens of a script, we can examine the social consequences and meanings of technologies such as KOMP [15]. This includes the practices and perspectives of different characters, their needs, and how roles, responsibilities, and relations between different actors are distributed.

## Methods

### Research design

This study examines the improvised use of a new interactive technology during the pandemic in care facilities. Since there is a scarcity of knowledge about the use of interactive technology for social communication in care facilities [20], it is appropriate to adopt a qualitative, exploratory design to investigate how and why health care professionals facilitate communication and social engagement in care facilities. Here, data triangulation, where more than one method is used to collect data on the same phenomenon, was necessary to capture a range of relevant dimensions about the use of KOMP and to ensure valid interpretations of the data [21].

First, focus group interviews with a moderated dialogue helped us obtain data from a group of health care professionals experienced with KOMP and to gain an in-depth understanding of attitudes and sentiments towards the technology [22, 23]. Participants were strategically sampled for the interviews to include a variety of professional perspectives on the use of interactive technology in care facilities. However, what people say in the context of an interview and what they do in real-life practice may differ in profound ways. We therefore conducted systematic field observations in a short-term care facility to explore how KOMP was performed ‘in the wild’ [24]. Fieldwork to document situated practices in the natural setting of the care facility was supported by individual interviews with health care professionals to gain insight into perceptions, experiences, and beliefs about KOMP [25].

### Sampling

Recruitment for the focus groups began in August 2020. The first and last authors had a discussion with the person in charge of assistive living technology at the municipality to get information about which care facilities have experience with the use of KOMP. Based on this, we sent emails to the managers of 16 short-term and long-term care facilities that had recently made use of KOMP and distributed in different geographical locations in the same county. The emails included the study description, an invitation to participate, a consent form and contact information for the first and the last authors. Managers then forwarded emails to health care professionals in their care facilities. From these 16, we received eight positive answers. Among them, one care facility agreed to participate in both the interviews and observations, and one care facility apologised for withdrawing before the interview, due to conflicting commitments. From the remaining seven care facilities we recruited a total of 12 participants, organized in three focus groups (two groups had five participants, and one group had two). One focus group consisted of five participants from the care facility where the observations were conducted. The other two focus groups included seven participants from different care facilities. Participants in the focus groups counted eight registered nurses (six of them were health care managers), one radiographer, two assistant nurses and a physiotherapist.

Additionally, we obtained permission to make observations in a care facility that provided residents with different AAL devices, including KOMP. Observations were made over six days in November 2020. Ten health professionals, including five registered nurses, two assistant nurses, two care facility doctors, and an activity manager, agreed to participate in the observational study and share their experiences.

In total, the study included 22 health care professionals, 18 females and four males. Their ages ranged from 26 to 60 years. Their experience with health care work ranged from one year to three decades. For details, see Table 1, which follows guidelines for reporting qualitative research [26].

**Table 1:** Overview of the participants

Number	Profession	Individual interview	Focus group	Gender
01	AN		FG1	F
02	HM		FG1	F
03	HM	**×**	FG1	F
04	RN		FG1	F
05	RN		FG1	F
06	HM		FG2	F
07	HM		FG2	F
08	HM		FG2	F
09	HM		FG2	M
10	HM		FG2	M
11	AN		FG3	F
12	PH		FG3	F
13	AM	**×**		F
14	RN	**×**		F
15	RN	**×**		F
16	RN	**×**		F
17	RN	**×**		F
18	RN	**×**		F
19	AN	**×**		F
20	AN	**×**		M
21	MD	**×**		F
22	MD	**×**		M

*Abbreviations*: *RN* registered nurse, *AN* assistant nurse, *AM* activity manager, *PH* physiotherapist, *MD* medical doctor, *HM* health care manager, *FG* focus group

### Setting

The care facility where we performed observations had two wards for short-term stays, where residents would live from two to eight weeks (notably, due to long waiting lists in long-term care, some residents had their stay extended well past eight weeks). Observations were made during the morning shift, where there were six or seven health care professionals at work. Among the 31 comorbid residents in these two wards, eleven residents used the KOMP from 2019 to 2020. A majority of these residents had reduced cognitive ability, with Mjørud et al. [27] estimating this share in care facilities to be approximately 80%.

Recruitment of health care professionals was based on their experiences with residents who had used KOMP. Notably, this particular facility had introduced an assortment of technologies to support the care of residents over the past years, including systems for remote monitoring of various parameters and behaviours, including mobile phones and iPads, and sensor-based devices such as RoomMate [28], and Somnofy [29]. However, most of the care facilities in the municipality used other devices, such as smartphones and tablets. These devices, however, were not considered to be suitable for older people, especially those with hearing and vision impairment, and problems with capacitive sensing. To efficiently use these devices, older people were said to need assistance from the health care staff.

KOMP had also been used by a few residents before the pandemic. As a result of their ongoing efforts to adopt new technologies for care, this facility employed a facilitator who trained staff in their use and fixed technical issues. Furthermore, it should also be noted that focus group interviews were not restricted to staff from this facility and included health care professionals from other short- and long-term care facilities to gain insight into different perspectives on KOMP use.

### KOMP

**Fig. 1:**
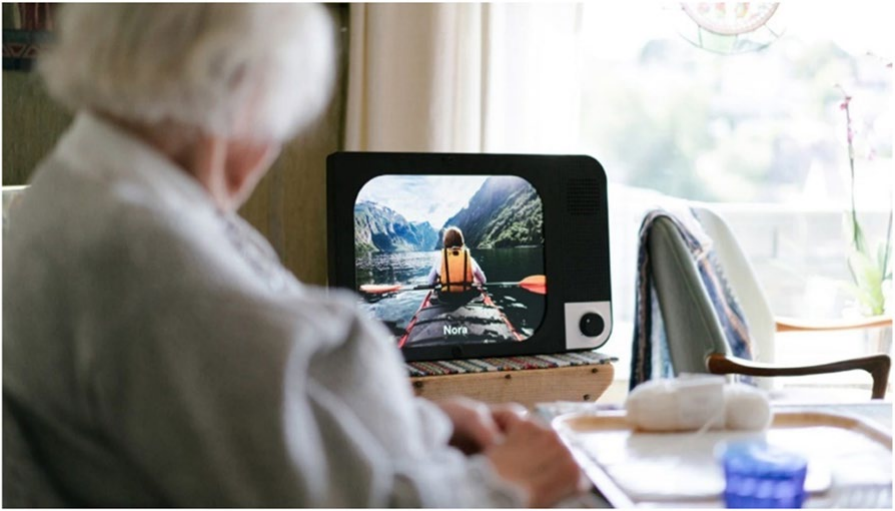
KOMP from No Isolation (©Photographer Estera K. Johnsrud). The image is accompanied by a caption (white text) describing the name of the person who has sent the image (here: ‘Nora’)

KOMP, as shown in Fig. 1, is an interactive tool designed to connect elderly individuals with their families and friends via pictures, text messages, and video calls. KOMP is marketed as the “one-button computer connecting generations” and designed to look like a small TV with a 17-inch screen. It has a Wi-Fi connection and an eight-megapixel camera. Intentionally designed to have a simple user interface, with a single on/off knob on the front, KOMP is advertised as not requiring any complicated training or effort to use by elderly individuals. The screen is used to display images either through a live video feed or as a series of rotating still images with accompanying text in large fonts. The device does not rely on a touch screen as an interface to avoid problems with capacitive sensing among elderly individuals (see black knob in lower right corner). KOMP is also designed to broadcast sound clearly and loudly [30].

The care facilities use a commercial version, known as KOMP PRO, designed for communication between residents and their next of kin, as well as between residents and health care professionals. Despite KOMP’s simple physical interface, next-of-kin and health professionals primarily interact with the device through an app that is downloaded to a phone or tablet device. Having set up a user profile through this application, it is possible to send images and texts, and make video calls directly to the KOMP with a passcode or invitational code unique to that device.

In Akrich [14] terms, designers of such devices rely on a “projected user” with specific competencies to productively use the technology. In the case of KOMP, the device was originally designed to be used by independent older persons who had adequate cognitive and physical abilities and lived at home [30]. As described by its manufacturer No Isolation, the device “bridges the communication gap between those that struggle to use modern-day technology, and their more tech-savvy family and friends”. However, our analysis will highlight how productive applications of KOMP in the context of care facilities require considerable efforts to “re-script” the technology, in Akrich’s terms.

### Data collection

Data collection began in September and lasted throughout November 2020. The first focus group interview was carried out in person at the meeting room in the same care facility where observations were conducted. Because of restrictions pertaining to COVID-19, the next two focus group interviews were arranged virtually in October 2020 (using Microsoft Teams).

Participant observations were performed in two wards in the short-term care facility in November 2020 for three days in each ward during the morning shift. These observations were documented through comprehensive fieldnotes. Additionally, 11 individual interviews were carried out.

 Both the focus group interviews, and the individual interviews were supported by a semi structured interview guide based on open-ended questions. The goal was to generate in-depth responses from informants about their experiences and perceptions of using interactive technologies such as KOMP in their work. Questions deliberatively focused on the impact and challenges of using interactive technology in daily life and social activities to support well-being and address social isolation. During interviews, the guide was complemented by follow-up questions to pursue additional topics that appeared through the conversation at the interviewers’ discretion. A moderator (first author) and a secretary (last author) conducted the focus group interviews, modelled after Patton [21]. The moderator hosted the interviews, while the secretary took notes and regularly summed up the contents to validate intended meanings throughout the session. Focus groups lasted 60 and 90 min. Individual interviews with health care professionals were conducted by the first author, lasting between eight and 24 min, with an average of 14 min. All interviews were digitally recorded and then transcribed verbatim in Norwegian.

 Field observations at the care facility were carried out by the first author. Her role as a researcher was disclosed to all participants in the study [31]. Having negotiated her status in the field around her health care background as a physician now researching the use of KOMP in caring practices, the first author was ‘marginally involved’ in work and conversations at the facility [21]. Descriptive fieldnotes from these observational sessions were transcribed in Norwegian.

### Data analysis

Answering how and why health care professionals facilitate communication and social engagement between older people and their next of kin in care facilities during a period of rapid increase in the use of interactive technologies required an inductive, qualitative approach to content analysis [32, 33]. Interviews and observational fieldnotes were transcribed as digital text, indexed, and then organized as separate files. Documents were then eligible for inductive, open coding to create higher-level categories and abstractions. The analysis was an outcome of three phases: preparation, coding and organization, and reporting. The preparation phase involved re-reading the material several times by the first and last authors to become familiar with the data. Raw data were then systematically organized by identifying and selecting meaningful units and labelling these units as substantial codes. This process of open coding of the materials from interviews and observations generated a total of 162 initial codes. Labelled codes were then inspected to ensure they reflected relevant aspects of the phenomena in question and the relevant units of meaning checked for consistency vis-a-vis each other. Following the framework of Elo and Kyngas [32], we then grouped the initial codes under higher-order headings based on content similarities, producing a total of 20 subcategories.

**Fig. 2:**
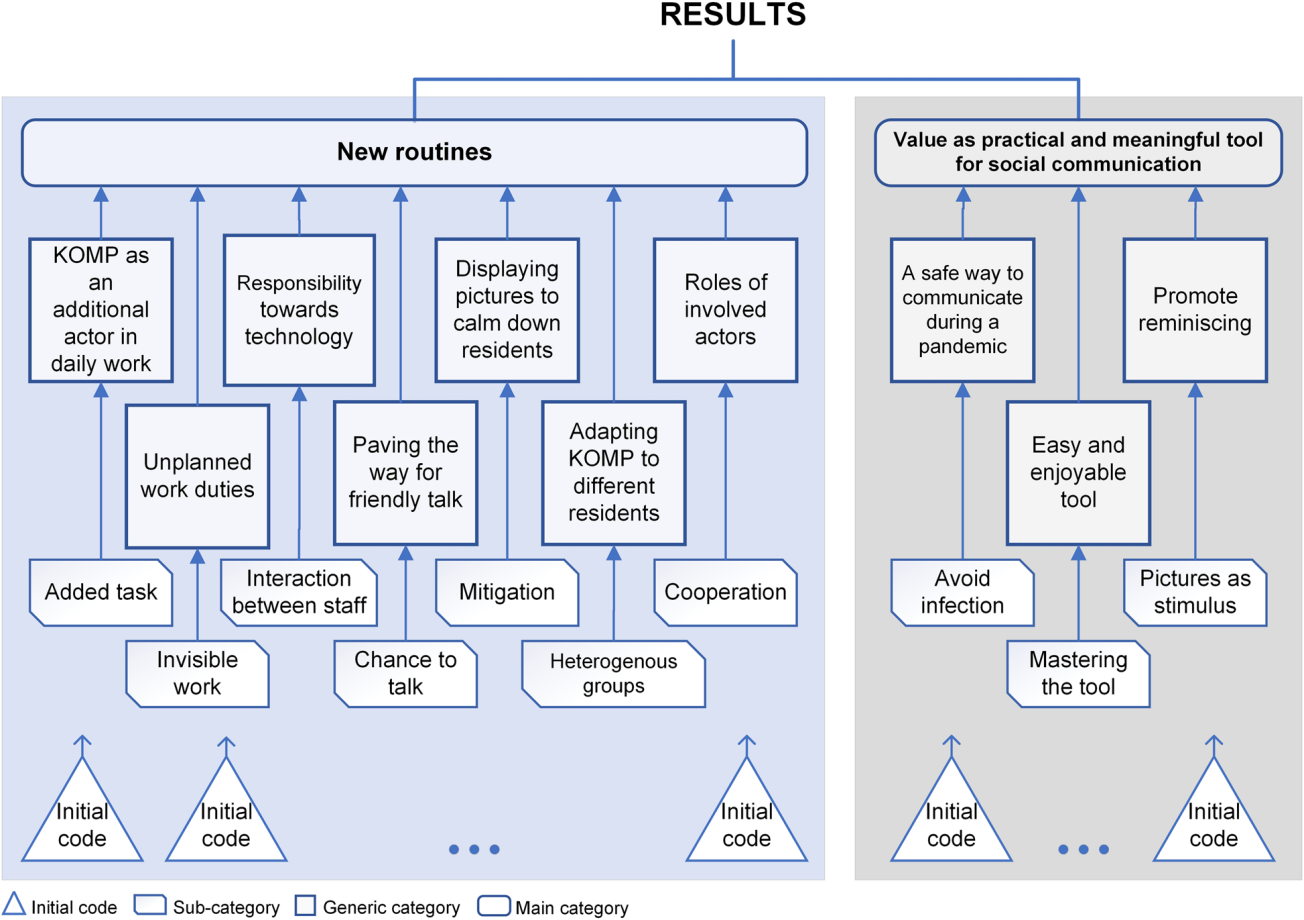
Schematic arrangement of codes, with a selection of ten sub-categories (out of 20 in total), ten generic categories, and two main categories, as revealed by content analysis. Examples from the 162 initial codes are not shown in the figure

Examples of these 20 subcategories are shown in Table 2, to exemplify the iterative coding process. Subcategories were then grouped together, yielding 10 generic categories. Examples of the most relevant subcategories and the generic categories are shown in Fig. 2. These generic categories were then grouped and condensed into two main categories that describe new routines accompanying the use of KOMP as an interactive technology and why KOMP is valuable, i.e., as a practical and meaningful tool for social communication. The coding process was supported by NVivo 12 (version 1.3). Using MindManager, we also created radial maps to identify, abstract, and group connections into broader, higher-level categories.

**Table 2:** Three examples from the abstraction process, from raw data to main analytical category

Quotes	Initial code	Sub-category	Generic category	Main category
“It has become a part of everyday affairs, or in a way a part of when the patient arrives, if he is capable of having a KOMP, he gets the offer, so it has become part of the care process”(PN14).	Part of care process	Added task	KOMP as an additional actor in daily work	New routines
“We have to rethink and be conscious about these sudden events that can be a problem” (PN02).	Technical issues	Interaction between staff	Responsibility towards technology	New routines
“When they get up in the morning, the first thing they do is to turn on the KOMP and look for new messages from their daughter or their sons, new images when they are at the cinema, or trips or in the mountains. There are a lot of residents who really enjoy this” (PN18).	Daily habit every morning	Mastering the tool	Easy and enjoyable tool	Value as practical and meaningful tool for social communication

*Abbreviations*: *PN* participant number

The materials coded under the main category of ‘new routines’ address the question of what health care professionals do to facilitate communication and social engagement between the residents and their next of kin through interactive technology in care facilities, and how this is accomplished. Materials labelled under KOMP’s value as a practical and meaningful tool for social communication help answer the question of why health care professionals use the technology to support communication and social engagement between the residents and their next of kin. Relationships among different levels in the coding scheme are displayed in Fig. 2.

## Results

### New routines

The coding process revealed seven categories about the prompt use of KOMP, which specify new work routines in the care facility associated with the technology.

#### KOMP as an additional actor in daily life

Field observations in November 2020 revealed that KOMP had become a part of the daily routine at the care facility. Its use was repeatedly mentioned and discussed in the ward, and yellow sticky notes about scheduled calls between residents and their relatives could be seen displayed around the nurses’ offices. The care facility had eight KOMP devices that rotated between residents, with appropriate measures for infection control, in between. In addition, several devices had been bought privately for residents by their next of kin. Participants in the study considered KOMP to have become an integrated part of caring practices in the facility. As one nurse described, “It has become a part of everyday affairs, or in a way, a part of when the patient arrives. If he is capable of having a KOMP, he gets the offer, so it has become part of the care process” (Participant Number,PN14). Staff reported that they had residents who made good use of KOMP and that there was a need to facilitate its use. “We have one patient who has the screen in her room during breakfast and then enjoys the photos and answers calls from next-of-kin who call” (PN01), a nurse reported. To initiate a video conversation when the KOMP rang, staff moved residents from the common room to their private rooms. However, facilitating these video communications also required staff to frequently answer calls and text messages from relatives in advance to coordinate conversations with KOMP. Field observations also revealed that health care professionals developed routines to encourage residents to communicate more frequently with their relatives using the KOMP. However, although there were attempts to involve most residents in social activities through KOMP, some residents also had challenges that could not be easily accommodated with the technology, such as reduced physical and cognitive capabilities that prevented them from productively using KOMP as a communication device.

#### Unplanned work duties

The health care professionals stated that KOMP requires a type of “planning” that was not readily identified as such in the regular sense of the term. For example, when a resident failed to use KOMP without additional support, relatives would call one of the caregivers to arrange and facilitate a conversation. One of the caregivers would then turn on the KOMP while also ensuring that the resident’s door was kept open to make certain that the resident was engaging with the caller during the duration of the call before turning it off. Some nurses experienced that this calling routine required considerable time and effort. They added that even in the case of patients who managed to initiate calls with KOMP themselves, relatives often failed to get the reply they expected when dialling the KOMP directly, upon which they would call the staff on the phone to ensure that the resident was doing well.

#### Responsibility towards technology

Health care professionals reported that they were committed to using interactive AAL technologies during the pandemic. However, new responsibilities with respect to the technology also added new routines to staff workloads. For instance, staff would always test new digital tools before using them in the ward, to ensure usability and feasibility and to mitigate problems such as poor Wi-Fi reception or other technical issues. “We have to rethink and be conscious about these sudden events that can be a problem” (PN02), one nurse reported. The participants noted that using the technology was often complicated by unforeseen events, such as missing codes and passwords, low battery notifications, and messages on private phones from relatives reminding the staff to turn on or off a given KOMP. Occasionally, the device would also call out loudly while health care professionals were in the middle of other tasks, meaning that they had to rush off to answer the device to keep the ward quiet. As one nurse reported, “After finishing a video call, we must remember to turn off KOMP to limit interruptions while we are doing other tasks and to keep it, the ward, quiet” (PN03).

#### Paving the way for friendly talk

A main feature of KOMP is the display of a photo library with images sent from next of kin. To communicate with the KOMP, an application for a smartphone or tablet needs to be installed, and a user profile is created. It is then possible to add other family members so that they can also communicate with the device via the app. One popular form of communication was sending family photos or other images that the resident might be interested in. Images in the photo library are rotated as still images on the large screen over a certain number of days. Health care staff appreciated how KOMP displayed photos in this manner, since this provided them with a resource for friendly discussions about the resident’s family, such as specific memories that the elderly recalled when looking at the display. When visiting the private rooms of patients, the array of photos displayed on the KOMP from next of kin frequently became ‘talking points’ that invited meaningful conversations between residents and staff.

#### Displaying pictures to calm down residents

Occasionally, nurses and relatives collaborate to calm down agitated residents by strategically making use of the KOMP. For instance, staff reported that they sometimes displayed specific pictures, which they assumed had a relaxing effect on the behaviour of residents who acted confused and restless. They would, on occasion, obtain information from relatives about specific preferences for pictures. Caregivers also reported that some residents were noticeably calmer after KOMP sessions with their families. As one nurse pronounced, in plain terms, “daily conversation with a daughter or a spouse with KOMP can improve a resident’s mood” (PN01, 03). Staff also noticed that some residents who would walk restlessly through corridors in the facility would sometimes remain seated for longer time periods to enjoy the pictures displayed on the screen. They also exemplified how one patient suffering from impaired vision preferred to contact family via KOMP rather than engage the family through other social activities, such as outside strolls. This preference was attributed to the resident’s worry over the inability to orient in outside surroundings.

#### Adapting KOMP to different residents

KOMP is not suitable for all residents and situations. Depending on their cognitive and physical abilities, many residents require help for even very simple tasks, such as turning on and off the device, despite its one-button interface. Nearly all participants reported that cognitive disorders such as dementia generally made social interactions challenging, adding that it was necessary to always map out the needs, benefits, and risks of using technology with different residents.

 A physician at the care facility highlighted the unabating importance of bodily function for social interactions with KOMP, despite the apparent simplicity of the device’s interface. This point can be illustrated by an observation from an assistant nurse, who described how one resident with impaired vision had dropped KOMP on the floor several times, simply because she could not see where it was placed. As such, even a deceptively simple device such as the KOMP, which is designed to require little to no effort to operate, has an embodied character. In the observed facility, for instance, only two out of 11 patients had adequate physical and cognitive abilities for turning their KOMP on without assistance from staff. This aspect is not trivial since it has implications for caregiver workload at the facility, as staff sometimes have to service multiple residents and their next of kin while struggling to make the technology work for their respective conversations.

#### Roles of involved actors

Facilitating the use of KOMP required a cast of characters that included residents, relatives, health care professionals, and technology facilitators, all with different experiences, roles, and expectations. Relatives send and update the photo library to involve their loved ones in their lives. In the words of one health care professional, “relatives are good at updating KOMP with interesting pictures so residents can feel that they share the moments of their relatives” (PN08). Health care staff reported that this made residents feel more actively engaged in the daily lives of their families. Some relatives also preferred to purchase a KOMP device privately to have more freedom to communicate. This was beneficial for other residents since it meant fewer users per shared device and reduced the workload of health care staff who would otherwise spend valuable time administering different user accounts. Health care staff also reported how relatives played a key role during the pandemic by adopting KOMP as a safe alternative to physical visits, in compliance with strict measures for infection control. In the words of one professional, “with KOMP, relatives become more available than before, they can call daily or many times a day” (PN15). Observations, however, revealed that health care professionals were more comfortable using KOMP when a technology facilitator was available. In the words of one nurse, “as long as we have a person who helps and facilitates with technology, it works well…” (PN16). From her perspective, the technology facilitator played a significant supportive role in the daily implementation of interactive technology by answering questions about the technology from staff and troubleshooting unexpected technical issues.

### Value as practical and meaningful tool for social communication

#### A safe way to communicate during a pandemic

 In interviews, participants stressed that COVID-19 triggered an increase in KOMP use, as the technology offered a means to safely maintain communications despite mandatory measures for social distancing between residents and next of kin. Staff added that KOMP helped residents maintain contact with children, grandchildren, and family members, despite considerable physical distance. Health staff disclosed that families who used KOMP to communicate with residents were generally satisfied, as it helped them to see each other more frequently. Notably, they suggested that KOMP’s video functionality and photo libraries helped some patients with dementia to better recognize and connect with their relatives. Some of these patients could sometimes spend many days without family visits, and KOMP afforded other kinds of embodied interaction than what is possible over the telephone. Not only were there strict restrictions on physical visits during the pandemic, but these required prior planning, including booking an appointment for a limited duration at a safe location in the facility to reduce the risk of viral transmission. In comparison, digital visits made it possible for some residents to engage in video communications several times per day, although the availability of next of kin and staff capacity to some extent constrained the frequency of digital meetings.

#### Easy and enjoyable tool

Staff reported that KOMP’s user interface was easy to use. Compared to a smartphone or tablet, for instance, the device has a large screen and a single knob, both convenient for vision-impaired users. As one nurse disclosed, KOMP was now integrated into the daily routines for many residents who enjoy using it for social connectedness: “When they get up in the morning, the first thing they do is to turn on the KOMP and look for new messages from their daughter or their sons…. there are many residents who truly enjoy this” (PN18). In terms of user issues, in the narrow sense of problems pertaining to the interface *per se*, these appeared limited to the cognitive and physical challenges of specific residents.

#### Promote reminiscing

Health care professionals described how KOMP supported various forms of ‘memory work’, a sustained effort by staff to stimulate memory and recall by jointly watching pictures on the KOMP while talking about these visuals with the resident. Noting that residents were often homesick, staff suggested that KOMP offered a medium by which they could jointly look at pictures, recall significant events in their lives, and talk about meaningful events. Caregivers suggested that this imagery helped keep residents socially connected and stimulated the recall and articulation of past experiences. As one nurse remarked, “looking at old family photos, birthplaces, and the places where they grew up, refreshes their memory” (PN04). Caregivers also observed that KOMP offered an opportunity to share significant events with family members, such as gardening work, dinner preparations, and birthday celebrations. This ‘memory work’ included talking about pictures of significant others, such as grandchildren and other categories of kin, which some residents had problems recognizing.

## Discussion

Studies on the long-term implementation and feasibility of digital interventions for social communication in care facilities are still limited due to restricted access, challenges with recruiting of elderly users, and ethical challenges [34, 35]. Other studies have shown that residents in care facilities are less likely to utilize interactive compared with those living in private homes, as physical and cognitive limitations prevent them from employing such technologies without considerable support from health care professionals [36–38].

Asking the question of how and why health care professionals facilitate communication and social engagement between residents and their next of kin in care facilities using KOMP helps us better understand the contingent process by which novel technologies of care are implemented in the wild. The rapid adoption of KOMP at the care facility during the pandemic entailed new forms of collaboration between actors. Akrich’s notion of scripting and re-scripting casts light on key dimensions of collaborative efforts around this socially situated technology, whereby new routines emerged to facilitate implementation of KOMP. In the context of nursing practice, Zisberg et al. ([39], p. 446) have described routines as “a concept pertaining to strategically designed behavioural patterns (conscious and subconscious) used to organize and coordinate activities along the axes of time, duration, social and physical contexts, sequence and order”. In the case of KOMP, some of these additional routines manifested as highly visible tasks at both the individual and organizational levels, while others were partially obscured by other features of the everyday workload at the care facility. Some of these everyday routines were tailored to suit the needs of particular residents and their next of kin [40], while others entailed new organizational routines and responsibilities towards a technology that would, on occasion, act unpredictably [41]. An example of a new, visible routine was the assessment of whether it would be convenient to offer KOMP to newly admitted residents. On the other hand, an example of a partially obscured task was the considerable work necessary to facilitate video communications to residents who could not use KOMP without staff assistance. An implication of such coordination in advance of conversations is that the technology does not work as spontaneously as the script for “projected users” might suggest [14]. It is well known that adding such ‘invisible’ routines can be taxing on workers [40]. In the context of the nursing home, “re-scripting” a technical object such as the KOMP beyond the “projected user”, through new routines and creative adaptations that resonate with user needs in the wild, requires considerable work on behalf of health care professionals [14]. Participants in this study noticed how the introduction of KOMP as an interactive technology for supporting the social life of residents entailed a change in their professional responsibilities and their relationships with both residents and their families. In practice, this change in roles and responsibilities also demands a “re-scripting” of existing, routine work tasks [16]. Since novel interactive technologies may entail new tasks and additional labour, there is a need to investigate how these affect the working environment of health care professionals in the long term.

In this study, we have seen examples of how KOMP was introduced in care facilities during a pandemic characterized by strict measures for social distancing. Without much advance preparation or planning, this technology helped elderly residents maintain social communications and engagement with their next of kin. Studies recommend both the adaptation of existing technologies, as well as development of new technologies to address challenges associated with the COVID-19 pandemic [42].

As an example of inclusive technology design, KOMP’s projected users (again invoking one of Akrich [14] terms) are cognitively and physically capable of using the device independently. In the context of care facilities, however, where many residents suffer from cognitive decline and physical disability, there is a need for re-scripting, which entails new forms of work by health care professionals. For residents to benefit from KOMP, health care professionals had to adapt their work routines to fulfill individual needs while accommodating and balancing a variety of technological and social constraints.

The professionals reported that KOMP, as a novel interactive technology, demanded new responsibilities and commitments. According to Pols and Moser [15], technologies of care have normative implications by prescribing new relationships between different actors. These included preparations for unexpected technical faults, ensuring that users of KOMP did not disturb other residents, and attending to the intrusive demands of the technology despite having other important tasks to do. For some professionals, the use of KOMP also entailed a sense of moral responsibility, a “technological imperative” to utilize advanced technologies to enhance everyday life for residents at the facility [43]. Our results also suggest that while valuation is always determined contextually and directly influenced by expectations towards the service being provided [44], the added value of KOMP partly depends on the physical and cognitive abilities of each individual resident. Despite KOMP’s inclusive design, physical and cognitive disabilities can only be partially mitigated by organizational efforts and staff endeavours.

## Strengths and limitations

Data triangulation, combining focus group interviews with field observations and individual interviews, strengthens the reliability and internal consistency of this study. The purpose of triangulation was to gain insight into how KOMP was socially situated in care facilities and to assure sound interpretations of what KOMP meant for health care professionals. Observations of these professionals in the natural context of their workplace also helped the research team reach an ecologically valid understanding of how KOMP was integrated into daily practices of care and to identify challenges with the technology. Another strength is that the interlocutors in the study came from different professional backgrounds, thus offering a diverse set of perspectives on the use of interactive technology for elderly care. Limitations include a relatively limited period of participant observation in the short-term care facility. Long-term, immersive fieldwork would likely reveal other dimensions about the social significance of KOMP than those documented here.

## Conclusion

This study examined the ad hoc and prompt use of an interactive technology for social communication known as KOMP in care facilities during the pandemic through the conceptual lens of script theory [14]. The implementation of interactive technology for social engagement in care facilities is a complex process, as technologies do not work spontaneously on their own. Productive use of KOMP in care facilities required cooperation between a host of actors, including residents, relatives, health care professionals and technology facilitators. Despite its simple user interface, the use of KOMP is constrained by the physical and cognitive abilities of users. Not all users benefit from KOMP, and the process of rapidly implementing this interactive technology introduced new routine tasks and responsibilities for health care professionals. This process can be fruitfully analysed through the concept of “re-scripting”. In this process, technology and care are mutually shaped, as both are adapted to the needs and capabilities of different residents. Some of these new tasks were also partially obscured due to the complex nature of care work. The ‘hidden’ nature of tasks associated with this new interactive technology makes it difficult to estimate workloads and to evaluate the technology’s long-term feasibility. We suggest that health care managers should ask a deceptively simple question when introducing novel technologies in health care contexts, namely, what kind of ‘invisible’ work is entailed for health care workers by implementing the device in question? Answering this question will not be straightforward. More longitudinal research is needed to explore the long-term impact of using interactive technology in care facilities and how such technology adds value to the lives of residents and health care professionals.

## Data Availability

The datasets generated and analysed during the current study are not publicly available due to participants’ confidentiality but are available from the corresponding author on reasonable request.

## Abbreviations

AAL: Active-Assisted Living
NSD: Norsk Senter for Forskningsdata (Norwegian Centre for Research Data)
COVID-19: Corona Virus Disease of 2019
PN: Participant Number
